# Identification of a Novel CD8^+^ T cell exhaustion-related gene signature for predicting survival in hepatocellular carcinoma

**DOI:** 10.1186/s12885-023-11648-x

**Published:** 2023-12-04

**Authors:** Kejun Liu, Junhao Liu, Xusheng Zhang, Di Liu, Weijie Yao, Yang Bu, Bendong Chen

**Affiliations:** 1 Department of Hepatobiliary Surgery, General Hospital of Ningxia Medical University, Yinchuan, 750004 China; 2 School of Clinical Medicine, Ningxia Medical University, Yinchuan, 750004 China; 3 Department of Hepatobiliary Surgery, People’s Hospital of Ningxia Hui Autonomous Region, Yinchuan, 750002 China

**Keywords:** Hepatocellular carcinoma, CD8^+^ T Cell Exhaustion, Prognosis, LASSO regression analysis

## Abstract

**Background:**

Hepatocellular carcinoma (HCC) is a major health concern, necessitating a deeper understanding of its prognosis and underlying mechanisms. This study aimed to investigate the mechanism and prognostic value of CD8^+^ T Cell exhaustion (CD8^+^ TEX)-related genes in HCC and construct a survival prognosis prediction model for patients with HCC.

**Methods:**

CD8^+^ TEX-related genes associated with HCC prognosis were analysed and identified, and a prognostic prediction model was constructed using the ‘least absolute shrinkage and selection operator’ Cox regression model. Immunohistochemistry was used to verify the expression of the model genes in HCC tissues. A nomogram was constructed based on risk scores and clinical features, and its predictive efficacy was verified. The expression of *STAM*, *ANXA5*, and *MAD2L2* in HCC cell lines was detected by western blotting; subsequently, these genes were knocked down in HCC cell lines by small interfering RNA, and their effects on the proliferation and migration of HCC cell lines were detected by colony formation assay, cck8, wound healing, and transwell assays.

**Results:**

Six genes related to CD8^+^ TEX were included in the risk-prediction model. The prognosis of patients with HCC in the low-risk group was significantly better than that of those in the high-risk group. Cox regression analysis revealed that the risk score was an independent risk factor for the prognosis of patients with HCC. The differentially expressed genes in patients with high-risk HCC were mainly enriched in the nucleotide-binding oligomerization domain-containing protein-like receptor, hypoxia-inducible factor-1, and tumour programmed cell death protein (PD)-1/PD-L1 immune checkpoint pathways. The CD8^+^ TEX-related genes *STAM*, *ANXA5*, and *MAD2L2* were knocked down in HCC cell lines to significantly inhibit cell proliferation and migration. The prediction results of the nomogram based on the risk score showed a good fit and application value.

**Conclusion:**

The prediction model based on CD8^+^ TEX-related genes can predict the prognosis of HCC and provide a theoretical basis for the early identification of patients with poor HCC prognosis.

**Supplementary Information:**

The online version contains supplementary material available at 10.1186/s12885-023-11648-x.

## Background

Primary liver cancer is a malignant tumor with a high incidence and poor prognosis worldwide. Hepatocellular carcinoma (HCC) constitutes 85–90% of primary liver cancers, has a poor prognosis, and ranks as the second leading cause of cancer-related deaths in China, with only 12.1% of patients surviving 5 years. Over half of the global HCC cases and deaths occur in China [1, 2]. Additionally, the development of HCC involves complex interactions among various factors, pathways, and systems. Despite the helpfulness of staging systems such as Barcelona Liver Cancer Staging (BCLC), Chinese Liver Cancer Staging (CNLC), and tumour–node–metastasis (TNM) in assessing prognosis and outcome, the high heterogeneity of HCC leads to significant survival differences among patients at the same clinical stage [1]. Therefore, different classification and prognostic indicators are needed to facilitate individualised, comprehensive HCC treatment to further enhance patient survival.

Adaptive immune responses play a crucial role in preventing tumour occurrence and development [3]. CD8^+^ T cells are the main immune effector cells that mediate anti-tumour cell infiltration. However, continuous stimulation by tumour antigens causes infiltrating CD8^+^ T cells to eventually lose their effector functions and memory characteristics. This phenomenon, known as CD8^+^ T Cell Exhaustion (CD8^+^ TEX) [4, 5], results in a shift in functionality, such as diminished effector capabilities, sustained expression of inhibitory receptors, epigenetic and transcriptional profile changes, and metabolic alterations [6, 7]. CD8^+^ TEX poses a formidable obstacle to current anti-cancer immunotherapy, as they lose the ability to produce anti-tumour cytokines, along with compromised proliferation and cytotoxicity [8]. In patients with HCC, variations in prognosis may largely hinge on the extent of uncontrolled CD8^+^ T cell exhaustion [9, 10]. Previous studies indicate that CD8^+^ T cells in the intermediate stages of exhaustion exhibit the most favourable response to treatment with immune checkpoint inhibitors, which can effectively restore T cell cytotoxic functions [5, 11]. Therefore, a complementary diagnosis based on the CD8^+^ TEX stage (or CD8^+^ TEX subtype) is expected to positively enhance the therapeutic effectiveness of immune checkpoint inhibitors [12].

In this study, we comprehensively analysed CD8^+^ TEX-related genes prognostic for HCC and explored their value in predicting patient outcomes. We constructed a prognostic risk model for HCC and analysed and validated its predictive efficacy. The significance of screening and characterizing CD8^+^ TEX markers as well as potential HCC therapies, was analysed. Concurrently, the biological function of the CD8^+^ TEX-related genes in HCC was validated at the cellular level, providing new insights into individualized clinical treatment.

## Materials and methods

### Data sources

Ribonucleic acid sequencing (RNA-seq) data of 365 patients with HCC and their corresponding clinical information were downloaded from The Cancer Genome Atlas (TCGA; portal.gdc.cancer.gov) and Genotype-Tissue Expression (GTEx; gtexportal.org/home/) databases. Additionally, RNA-seq data and relevant clinical information from 240 Japanese patients with HCC were downloaded from the International Cancer Genome Consortium (ICGC) database to serve as an external validation cohort. Gene expression data were standardized using the ‘Sanger box’ tool (http://sangerbox.com/). Based on previous studies [13], specific pathways associated with CD8^+^ T cell exhaustion were identified, including ‘REACTOME_TNF_SIGNALING’, ‘REACTOME_INTERLEUKIN_2_SIGNALING’, and ‘REACTOME_INTERFERON_GAMMA_SIGNALING’ from the MsigDB database. The toxicity-related genes from the ‘KEGG_NATURAL_KILLER_CELL_MEDIATED_CYTOTOXICITY’ pathways were used as CD8^+^ T cell exhaustion-related genes, resulting in the acquisition of 270 T cell exhaustion-related genes. The network of the top 20 interacting proteins of model genes (Spearman correlation coefficient ≥ 0.9) was mapped using the String online database [14] in combination with Cytoscape (https://cytoscape.org) software. Furthermore, cBioPortal (cbioportal.org/) was used to analyse the mutation characteristics of the model genes in HCC based on the TCGA PanCancer Atlas [15].

### Liver cancer cell lines and experimental antibodies

MHCC97L, MHCC97H, HCCLM3, Huh-7, Hep3B, SK-Hep-1and LO2 cells were provided by the Hepatology Institute of Shanghai Fudan University, and SNU368 was obtained from the Korean Cell Line Bank (Seoul, Republic of Korea). Anti-*MAD2L2* (BM5428) and anti-*STAM* (A00864-1) antibodies were purchased from BosterBio (Pleasanton, CA, USA), while Anti-*TBL1XR1* (MBS850368), Anti-*ANXA5* (MBS474163), Anti-*FKBP1A* (MBS9404059) and Anti-*PPM1G* (MBS626576) antibodies were purchased from MyBioSource (San Diego, CA, USA).

### Immunohistochemical method

Post-operative paraffin tissue samples were collected from 20 patients with HCC who underwent surgery at our hospital between October 2021 and December 2022. The inclusion criteria were: postoperative pathological confirmation of hepatocellular carcinoma, no prior anti-cancer treatment before surgery, complete clinical data, and collection of both cancer tissue and adjacent normal liver tissue samples (> 2 cm away from the tumour). The exclusion criteria were: complications with other malignant tumours and co-occurrence of cardiovascular and respiratory diseases. The Ethics Committee of the General Hospital of Ningxia Medical University approved this study (No. KYLL-2022–0413). All study participants provided written informed consent.

The HCC tissue sections were de-waxed using xylene and an alcohol gradient. Rehydration was performed after 2 h in 60℃ ovens. After antigen repair, incubation at room temperature with 3% hydrogen peroxide was performed for 30 min. The samples were then immersed in phosphate-buffered saline for 1 h after vibration washing. Subsequently, the primary antibody was added and incubated at a constant temperature (4 ℃) overnight. The following day, the sections were allowed to reach room temperature, and secondary antibodies were applied after a vibration wash. They were placed at room temperature for 2 h. Finally, alcohol gradient dehydration, xylene transparency, and sealing were performed, followed by microscopic observation.

### Prognosis models for depleted CD8^+^ T cell-related genes

Differentially expressed genes (DEGs) were included in a univariate Cox regression analysis to identify prognostic genes with statistically significant differences. Subsequently, based on the R (R Foundation for Statistical Computing, Vienna, Austria) language package ‘glmnet’ [16], the DEGs related to HCC prognosis were analysed via LASSO regression with tenfold cross-validation. The model’s risk score was calculated for each patient using the coefficients from the least absolute shrinkage and selection operator (LASSO) regression analysis and each gene’s expression level. The risk score is computed as: ∑β_x_*Exp_x_ (β_x_ represents the coefficient of each gene screened by LASSO regression analysis, Exp_x_ represents the expression level of these genes). The median risk score was used as a cut-off value to divide the training set into high- and low-risk groups. Survival analysis was conducted by generating Kaplan–Meier curves using the ‘survival’ package, and time-dependent receiver operating characteristic (ROC) curves in the ‘time-ROC’ package were used to evaluate the predictive efficiency of 1-, 3-, and 5-year survival.

### Nomogram construction and verification

The RMS package [17] in R language software and Cox regression analysis were used to construct a column graph for prognosis prediction based on patient sex, age, Child–Pugh grade, alpha fetoprotein (AFP) level, pathological tissue grade, T stage, and risk score. Calibration curve and consistency index (C-index) were used to evaluate the validity of the nomogram.

### GO/KEGG enrichment analysis

The DAVID database (https://david.ncifcrf.gov/) was used for Gene Ontology (GO) function and Kyoto Encyclopedia of Genes and Genomes (KEGG) pathway enrichment analysis [18–21]. Gene function analysis included biological process (BP), molecular function (MF), and cellular component (CC), with a primary focus on KEGG pathways. The main enrichment results were visualized when the false discovery rate was < 0.05 and *P* < 0.05.

### Difference analysis of tumour immune microenvironment

The INESTIMATE algorithm was used to infer the matrix components and levels of immune cells, and tumour purity was estimated for each sample. CIBERSORT [22] is an analytical tool that uses gene expression data to estimate the composition of immune cells in a mixed-cell population. The components of the 22 immune cells in all samples were evaluated using the ‘CIBERSORT’ function package of the R software, and then the correlation between the risk scores of the prognostic model and the components of the 22 immune cells was analysed. Concurrently, the correlation between the risk score of the prognostic model and co-immune checkpoints was analysed.

### Drug sensitivity analysis

The ‘onco-Predict’ package was used to predict the treatment response to small molecular compounds in patients belonging to high- and low-risk groups. Drug sensitivity was predicted according to the half-maximal inhibitory concentration (IC50), and the differences in drug sensitivity between the high- and low-risk groups were evaluated. PubChem (https://pubchem.ncbi.nlm.nih.gov/) was used for visualising the 3D conformations of the drugs.

### Cell culture and transfection

All cells were cultured at 37 °C with 5% CO2 in Dulbecco’s Modified Eagle Medium (DMEM) medium containing 10% fetal bovine serum. The small interfering RNAs (siRNAs) targeting *STAM*, *ANXA5*, and *MAD2L2* were purchased from GenePharma (Shanghai, China). siRNAs were transfected into the MHCC97H, MHCC97L, and HCCLM3 cell lines using lipo2000. A follow-up experiment was conducted 48 h later.

#### Colony formation assay

A total of 3000 cells were evenly distributed in 6-well plates and incubated for 2 weeks. The cells were then fixed with 4% paraformaldehyde and stained with 0.5% crystal violet staining solution. The cell clusters were counted.

#### Cell proliferation assay

Cell viability was analysed using a cell counting kit-8 (CCK8) (Biomake, China) according to the manufacturer’s protocol. Cells were cultured in 96-well plates (3 × 10^3^ cells/well) and tested at 12, 24, 48, 72, and 96 h after plating. A volume of 100 μL CCK-8 working liquid (90 μL DMEM + 10 μL CCK-8 solution) was added to each well of the cells to be tested and cultured for 1 h. The absorbance at 450 nm was measured with an enzyme-labeled instrument.

#### Wound healing assay

Cells were cultured in 6-well plates. When the cell density reached 90%, the cell surface was marked with a 200μL plastic straw head and then they were cultured with serum-free DMEM medium. The scratches were photographed at different time points (0 h and 48 h) using an inverted microscope (Olympus, Tokyo, Japan), and cell migration was recorded.

#### Transwell assay

A transwell chamber with an 8μm aperture (Solarbio, Beijing, China) was used to detect migration capacity. For the cell migration experiment, 8 × 10^4^ cells were resuspended in 200 μL serum-free DMEM and placed in the upper chamber, and 600 μL DMEM containing 10% fetal bovine serum was placed in the lower chamber. The cells were cultured at 5% CO, and 37 °C for 48 h, and the upper chamber cells were wiped with a cotton swab. Cells in the lower chamber were fixed with 4% paraformaldehyde for 20 min and stained with 0.5% crystal violet for 30 min. The cells were photographed with a microscope in the upper left, lower left, upper right, lower right, and middle fields of view.

#### Western blotting

Cells were lysed with radio-immuno-precipitation assay (Beytime, Beijing, China), and then centrifuged at high speed at 4 °C to obtain the protein solution. The protein concentration was determined using the bicinchoninic acid (KeyGEN BioTECH, Jiangsu, China) method. Sample buffer was added and boiled for 10 min and stored at –20 °C for subsequent use. Proteins were isolated by sodium dodecyl sulfate–polyacrylamide gel electrophoresis. Proteins were then transferred to PVDF (Merck Millipore, Burlington, MA, USA) and incubated in 5% skim milk at room temperature for 1 h. PVDF membranes were incubated with a specific primary antibody at 4 °C overnight. After washing the PVDF membrane three times, a secondary antibody was incubated at 37 °C for 1 h, and the chemiluminescence method was used for detection.

#### Statistical analysis

All mRNA expression data were normalized and log-transformed using log2 (data + 1). Statistical analyses were performed using R software (V3.8.3). The Mann–Whitney U test was used to analyse the difference in expression between HCC and normal tissues, while the paired sample t-test was used for paired sample analysis (ns, *P* ≥ 0.05; *, *P* < 0.05; **, *P* < 0.01; ***, *P* < 0.001). The R package (ggplot2; version 3.3.3) was used for data visualization. The chi-square test or Fisher 's exact test was used to analyse the clinicopathological features of patients with low- and high-risk prognoses for liver cancer. The Kaplan–Meier method was used to compare the overall survival rate of patients with low- and high-risk prognoses for liver cancer, and the rank sum test was used to compare the differences between groups. Statistical significance was set at *P* < 0.05.

## Results

### Identification of DEGs of CD8^+^ TEX associated with prognosis in HCC

**Fig. 1:**
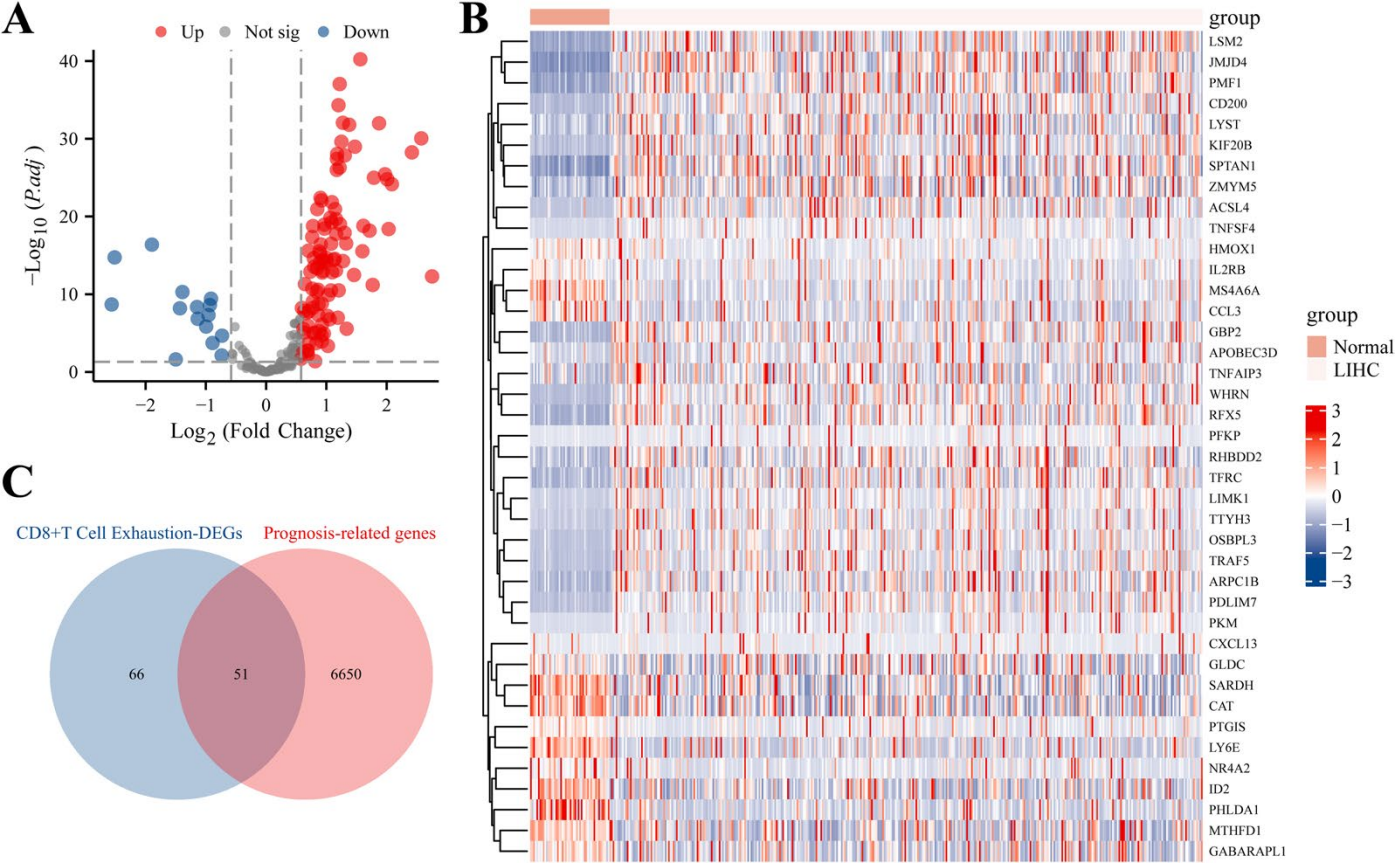
Identification of DEGs of CD8^+^ TEX associated with prognosis in HCC. **A** Volcano plot of DEGs of CD8^+^ TEX; **B** Expression heat map of top40-related DEGs of CD8^+^ TEX; **C** Venn diagram of DEGs of CD8^+^ TEX and genes associated with prognosis

The DEGs of CD8^+^ TEX between HCC and normal liver tissues were analysed based on the TCGA-LIHC dataset (Fig. 1). With |LogFC|> 0.58 and *P*.adj < 0.05 as the standard, 117 DEGs of CD8^+^ TEX were identified. Volcano and heat maps showed that 102 DEGs of CD8^+^ TEX were significantly down-regulated, while 15 DEGs of CD8^+^ TEX were up-regulated (Fig. 1A, B, Supplementary Table 1). Univariate Cox survival regression analyses of prognosis-related genes in HCC patients identified 6,701 HCC prognosis-related genes. After merging the DEGs of CD8^+^ TEX and prognosis-related genes, 51 candidate HCC prognosis-related CD8^+^ TEX-DEGs were obtained, and the results were visualized using a Venn diagram (Fig. 1C, Supplementary Table 2).

### Prognostic risk scoring model construction and verification

**Fig. 2:**
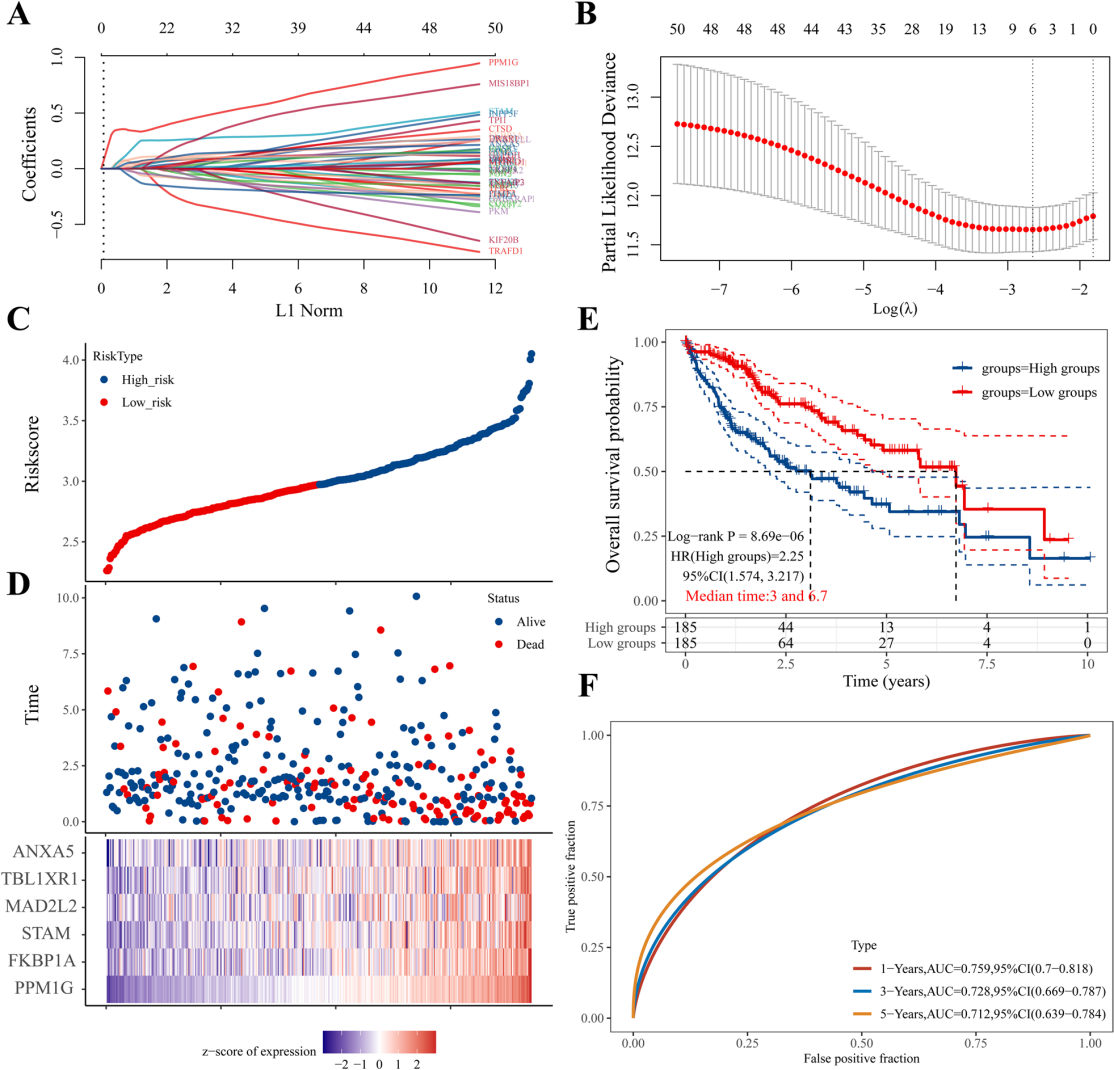
Construction of the prognostic risk model of CD8^+^ TEX marker genes. **A** Profile diagram of the distribution of Lasso regression coefficients; **B** tenfold cross-validation to select the optimal λ value; **C, D** Distribution of the median risk score of patients with HCC and its relationship with survival time; **E** Survival curve of patients with HCC; **F** ROC curve of the prognostic model

Based on univariate Cox regression, six genes (*PPM1G*, *FKBP1A*, *STAM*, *MAD2L2*, *TBL1XR1*, and *ANXA5*) were selected from 51 CD8^+^ TEX-related DEGs using LASSO regression analysis, and a prognostic risk score model of HCC was constructed (Fig. 2A, B). We constructed the prognostic risk score for patients with hepatocellular carcinoma as follows: risk score = (0.3519) × *PPM1G* + (0.0637) × *FKBP1A* + (0.0673) × *STAM* + (0.0373) × *MAD2L2* + (0.0031) × *TBL1XR1* + (0.0172) × *ANXA5*. Based on the median risk score (2.9732), all patients with HCC were divided into high- and low-risk groups (Fig. 2C, D). The results of the Kaplan–Meier survival curve analysis showed that the OS of the high-risk group was significantly lower than that of the low-risk group (Fig. 2E), and the difference was statistically significant (*P* < 0.05). The time-ROC curve showed that the model had good predictive efficiency (AUC > 0.7; Fig. 2F).

**Fig. 3:**
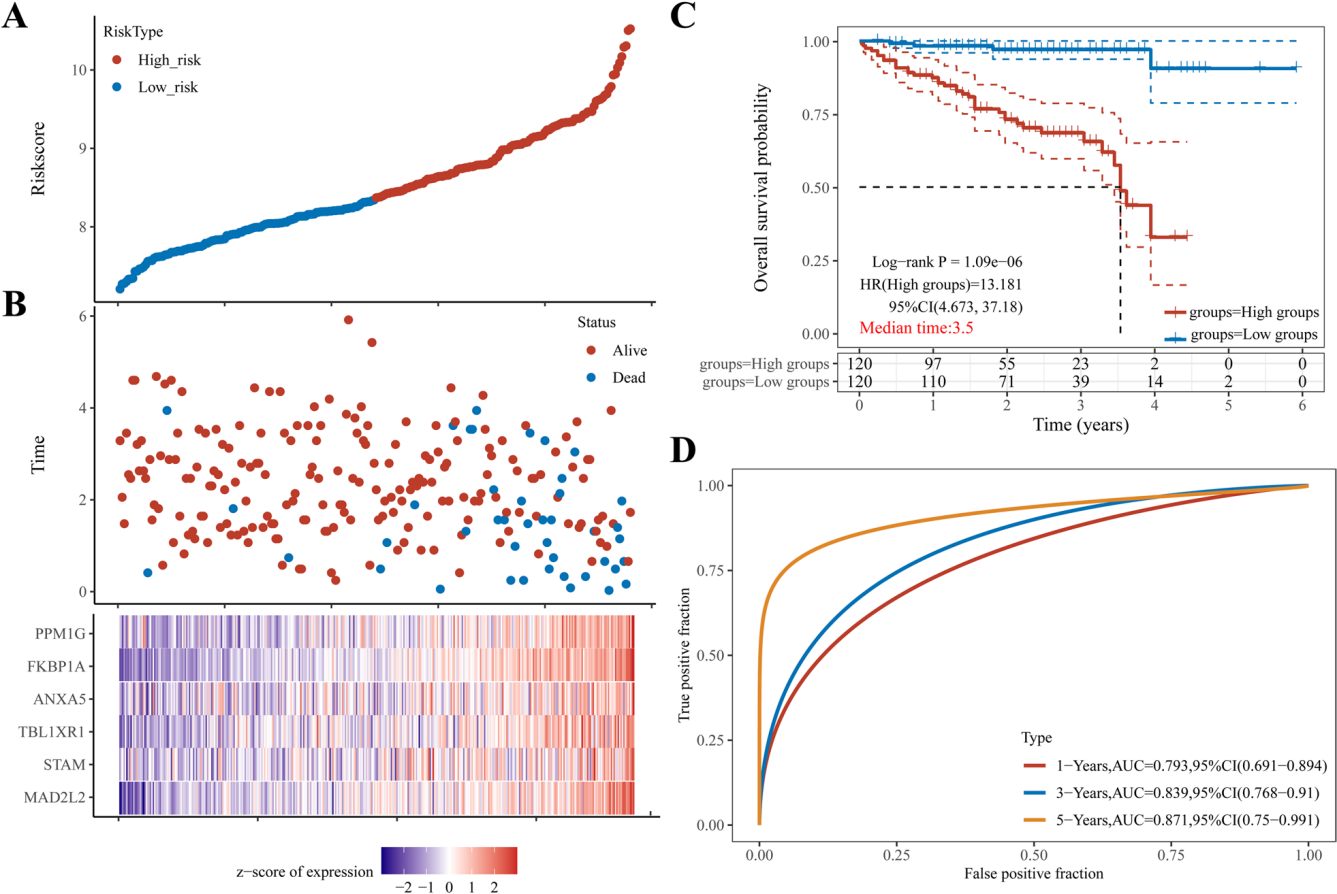
Validation of the prognostic risk model for CD8^+^ TEX marker genes. **A**-**B** Distribution of the median risk score of patients with HCC in the ICGC database and its relationship with survival time; **C** Survival curves of patients with HCC in the ICGC database; **D** ROC curve for prognostic model validation

The RNA-seq data and corresponding clinical information of 240 patients with HCC, used to validate the predictive model, were obtained from the ICGC database (Fig. 3). The data showed that patients in the low-risk category had a better prognosis (Fig. 3C), and the time-ROC curve confirmed the model’s strong predictive value for survival and prognosis (Fig. 3D).

### Gene expression and mutation in depleted CD8^+^ T cell-related prognostic risk model

**Fig. 4:**
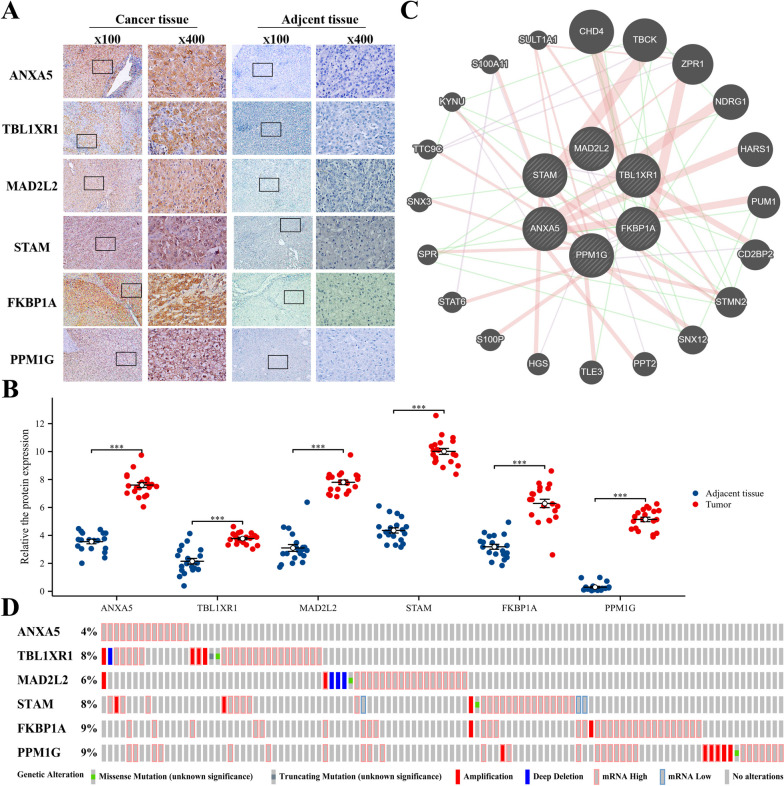
Expression and mutation of prognostic risk model genes in CD8^+^ TEX. **A** Expression of model genes in HCC tissues and adjacent tissues (SP method); **B** Expression of model genes in 20 cases of HCC tissues and adjacent tissues; **C** A total of the first 20 model genes were constructed. Protein–protein interaction network of expressed genes; **D** Genetic alteration of this model gene in HCC

The immunohistochemical analysis revealed that the expression of *ANXA5*, *TBL1XR1*, *MAD2L2*, *STAM*, *FKBP1A*, and *PPM1G* in HCC tissues of 20 patients was significantly higher than that in adjacent normal liver tissues (Fig. 4A, B) and was mainly located in the cytoplasm with brown granules. Protein interaction network analysis showed that the top 20 interacting proteins of the co-expressed genes in the model included *CHD4, TBCK, ZPR1. NDRG1, HARS1, PUM1, CD2BP2, STMN2, SNX12, PPT2, TLE3, HGS, S100P, STAT6, SPR, SNX3, TTC9C, KYNU, S100A11*, and *SULT1A1* (Fig. 4C). Simultaneously, cBioPortal was used to explore genetic changes in model genes in HCC. As shown in Fig. 4D, 108 (29%) of the 372 patients with HCC showed changes in the model genes. Notably, the *FKBP1A* and *PPM1G* genes in HCC displayed the highest alteration rates, with a 9% change rate. Additionally, the mRNA expression of these genes was markedly dominant.

### Correlation between prognostic risk score of depleted CD8^+^ T cells and clinicopathological features of patients with hepatocellular carcinoma

As shown in Table 1, significant differences were observed in BMI (*P* = 0.047), AFP level (*P* = 0.005), T stage (*P*** < **0.001), histological grade (*P*** = **0.002), pathological grade (*P* < 0.001), and microvascular invasion (*P* = 0.008) between the high and low-risk groups.

**Table 1:** Correlation between CD8^+^ TEX-related prognostic risk score and clinicopathologic features in patients with HCC

Characteristic	Total (N)	Risk Score		Statistic	*P-value*
		**Low (*n* = 184)**	**High (*n* = 181)**		
**Gender, n (%)**	**365**				
Female	119	59 (16.2%)	60 (16.4%)	0.01	0.913
Male	246	125 (34.2%)	121 (33.2%)		
**Age, n (%)**	365				
< = 60	173	81 (22.2%)	92 (25.2%)	1.43	0.231
> 60	192	103 (28.2%)	89 (24.4%)		
**BMI, n (%)**	332				
< = 25	175	79 (23.8%)	96 (28.9%)	3.96	**0.047**
> 25	157	89 (26.8%)	68 (20.5%)		
**AFP(ng/ml), n (%)**	276				
< = 400	213	123 (44.6%)	90 (32.6%)	7.97	**0.005**
> 400	63	23 (8.3%)	40 (14.5%)		
**Child–Pugh grade, n (%)**	237				
A	216	121 (51.1%)	95 (40.1%)	0.01	0.928
B	21	11 (4.6%)	10 (4.2%)		
**T stage, n (%)**	362				
T1	180	109 (30.1%)	71 (19.6%)	16.93	** < 0.001**
T2	91	39 (10.8%)	52 (14.4%)		
T3	78	29 (8%)	49 (13.5%)		
T4	13	4 (1.1%)	9 (2.5%)		
**Histologic grade, n (%)**	360				
G1	55	35 (9.7%)	20 (5.6%)	15.17	**0.002**
G2	175	96 (26.7%)	79 (21.9%)		
G3	118	48 (13.3%)	70 (19.4%)		
G4	12	2 (0.6%)	10 (2.8%)		
**Pathologic stage, n (%)**	341				
Stage I	170	105 (30.8%)	65 (19.1%)	**—**	** < 0.001**
Stage II	84	38 (11.1%)	46 (13.5%)		
Stage III	83	27 (7.9%)	56 (16.4%)		
Stage IV	4	3 (0.9%)	1 (0.3%)		
**Vascular invasion, n (%)**	311				
No	106	45 (14.5%)	61 (19.6%)	7.06	**0.008**
Yes	205	121 (38.9%)	84 (27%)		

### Prognostic value of risk score for predicting HCC prognosis

**Fig. 5:**
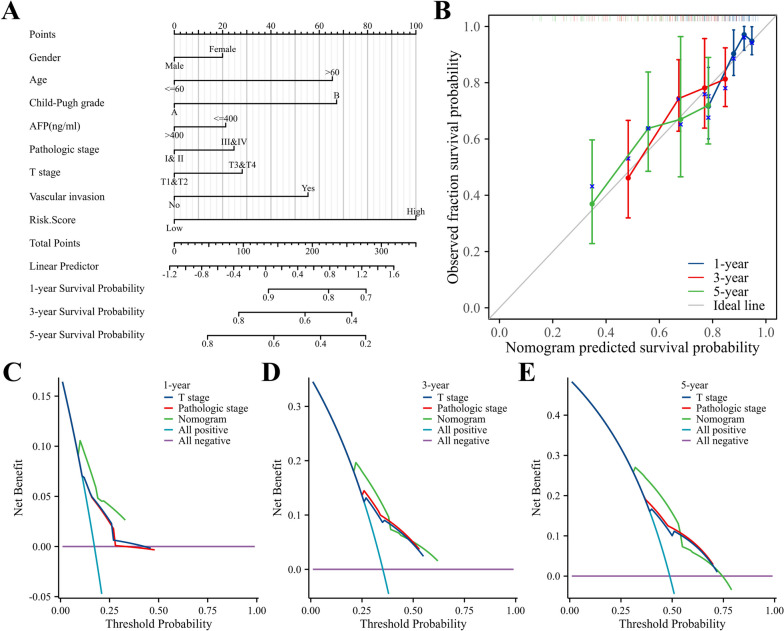
Construction and evaluation of prognostic nomogram for HCC. **A** A nomogram was established by integrating clinicopathological features and relative risk scores. **B** The prognostic calibration curve showed the predictive performance of the nomogram in the TCGA cohort. **C**-**F** 1-year, 3-year and 5-year decision curves

As shown in Table 2, COX regression analysis showed that the risk score (hazard ratio [HR] = 2.026, 95% confidence interval [CI]: 1.364–3.009, *P* < 0.001) was an independent risk factor affecting the prognosis of patients with HCC. Based on the risk score, various clinicopathological features (sex, age, Child grade, AFP level, pathological tissue grade, and T stage) were integrated to construct a nomogram (Fig. 5A). The nomogram’s predictive accuracy was validated through a line chart, demonstrating a C-index of 0.752 (95% CI: 0.721–0.802, *P* = 0.003; Fig. 5B). Finally, using the decision curve to compare the predictive value of the nomogram chart with the T stage and pathological stage, the net benefit was comparable, and the nomogram map outperformed the T stage and pathological stage in predicting the 1-year, 3-year, and 5-year survival rates (Fig. 5C–E).

**Table 2:** COX proportional risk model assessment of factors influencing survival prognosis in HCC patients

Characteristic	Total (N)	Univariate analysis			Multivariate analysis		
		**HR**	**95% CI**	***P-value***	**HR**	**95% CI**	***P-value***
**Gender(Female VS. Male)**	365	0.816	(0.573–1.163)	0.26			
**Age (< = 60 VS.60)**	365	1.248	(0.880–1.768)	0.214			
**AFP (ng/ml) (< = 400 VS.400)**	276	1.056	(0.646–1.727)	0.827			
**Child–Pugh grade (A VS. B)**	237	1.565	(0.743–3.297)	0.238			
**Vascular invasion(No VS.Yes)**	311	1.348	(0.890–2.042)	0.159			
**T stage(T1&T2 VS.T3&T4)**	362	2.534	(1.784–3.597)	** < 0.001**	1.738	(0.304–9.950)	0.534
**Pathologic stage (I& II VS.III&IV)**	341	2.449	(1.689–3.549)	** < 0.001**	1.178	(0.206–6.755)	0.854
**Risk Score(Low VS. High)**	365	2.375	(1.657–3.404)	** < 0.001**	2.026	(1.364–3.009)	** < 0.001**

### Exploration of gene regulation pathway in depleted CD8^+^ T cells of hepatocellular carcinoma

**Fig. 6:**
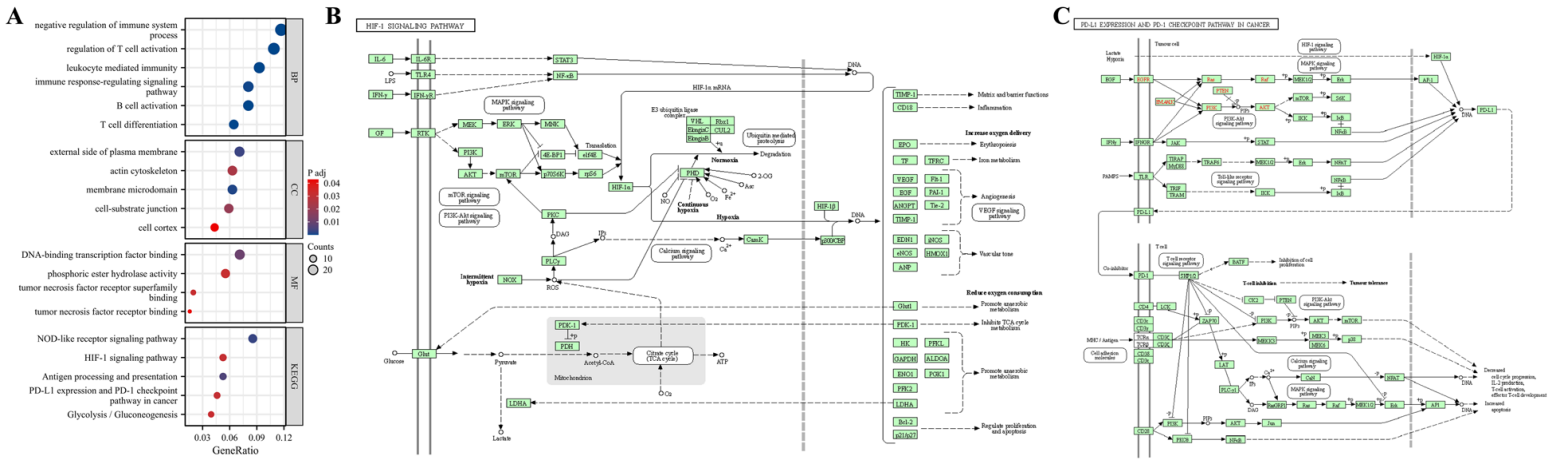
Differentially expressed gene enrichment analysis (DAVID) in high-risk HCC patients. **A** GO/KEGG pathway enrichment; **B** KEGG pathway: hypoxia-inducible factor-1 (HIF-1) signalling pathway; **C** KEGG pathway: tumour PD-1 / PD-L1 immune checkpoint pathway

As shown in Fig. 6, the biological signalling pathways related to differentially expressed genes in patients at high risk of HCC were analysed based on the enrichment of ClusterProfiler and ggplot2 packages in the R language. BPs were mainly associated with negative regulation of the immune system, regulation of T cell activation, leukocyte-mediated immune responses, immune response regulatory signal pathway, B cell activation, and T cell differentiation. CCs are mainly related to the lateral plasma membrane, actin cytoskeleton, membrane microdomains, cell–matrix junctions, and cell cortex. MFs were mainly enriched in DNA binding, transcription factor binding, phosphate hydrolase activity, and tumour necrosis factor receptor binding, while KEGG was mainly enriched in the NOD-like receptor signalling pathway, hypoxia-inducible factor-1 signalling pathway, antigen processing and presentation, tumour PD-1/PD-L1 immune checkpoint pathway, glycolysis/gluconeogenesis, and other signalling pathways.

### Evaluation of treatment response in high- and low-risk groups

**Fig. 7:**
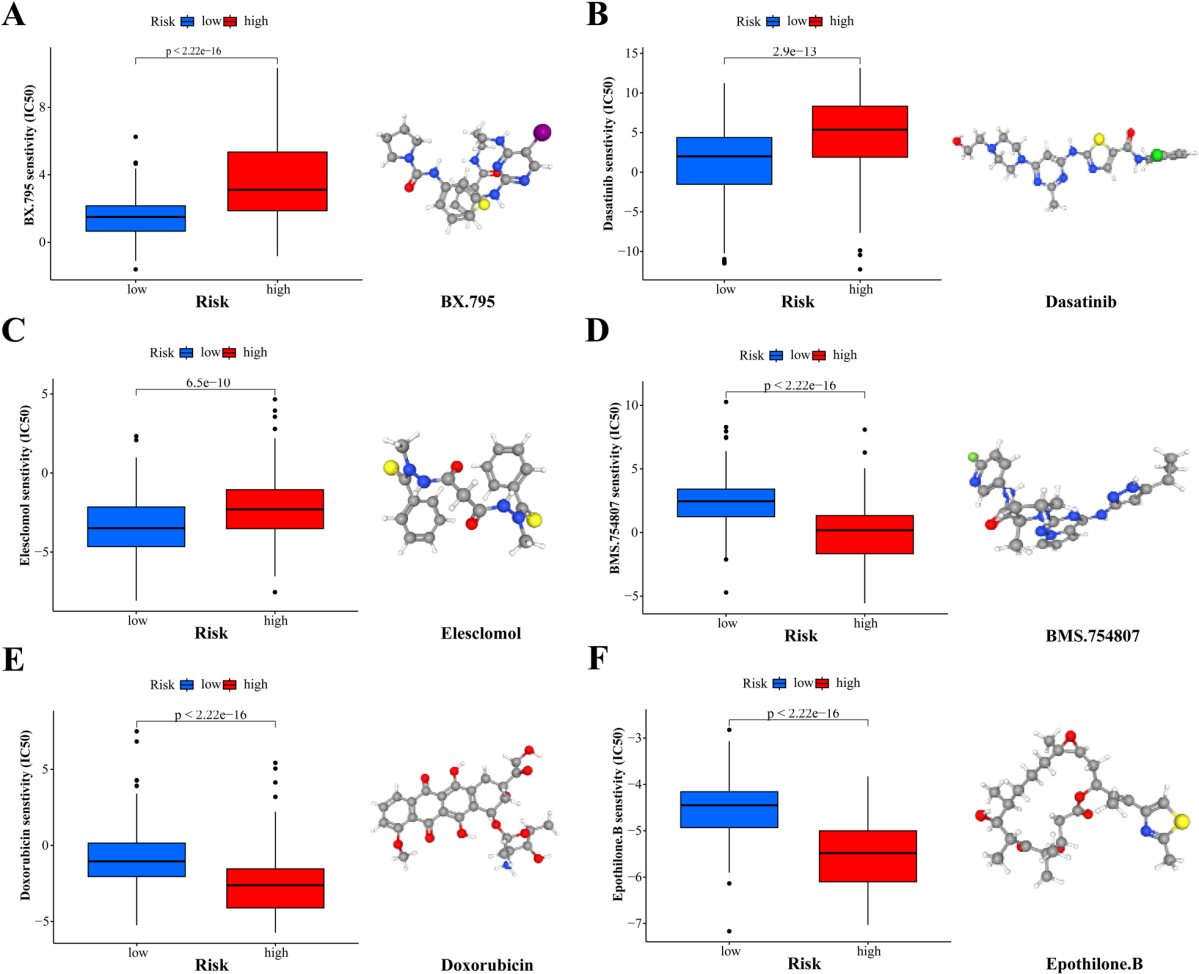
Screened drugs for the treatment of HCC. IC50 value and three-dimensional structure of BX.795 (**A**), Dasatinib (**B**), Elesclomol (**C**), BMS.754807 (**D**), Doxorubicin (**E**), Epothilone.B (**F**) in patients with high- and low-risk HCC

To predict the response to chemotherapy, we used a proportional algorithm to estimate the response to chemotherapy in patients with HCC based on the IC50 values available in the Cancer Drug Sensitivity Genomics (GDSC) database. In our study, 51 small molecule compounds with significantly different reactions were identified between the high- and low-risk groups (Supplementary Table 3). Patients in the low-risk group were more sensitive to BX.795, Dasatinib, and Elesclomol (Fig. 7A–C), whereas those in the high-risk group were more sensitive to BMS.754807, doxorubicin, and epothilone (Fig. 7D–F). The three-dimensional conformations of these four small-molecule compounds were visualized using PubChem.

### Knockdown of exhausted CD8^+^ T cell-related genes inhibited the proliferation and migration of HCC cells

**Fig. 8:**
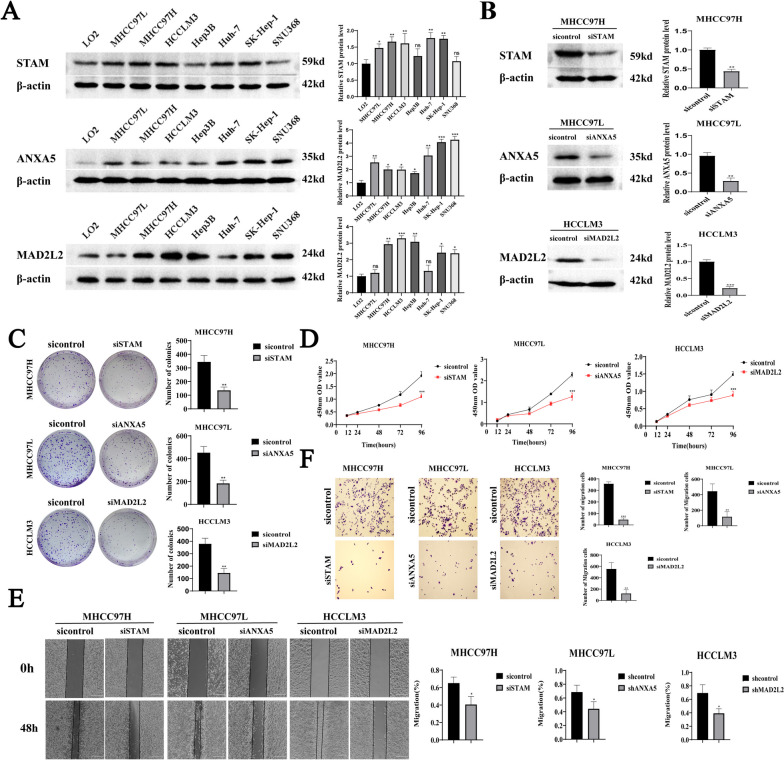
Expression of *STAM, ANXA5* and *MAD2L2* in HCC cell lines. **A** The expressions of *STAM, ANXA5* and *MAD2L2* in normal hepatocytes and hepatoma cell lines were detected using Western blotting. Knocking down *STAM, ANXA5* and *MAD2L2* inhibited the proliferation and migration of HCC cells. **B** The knockdown efficiency of *STAM, ANXA5* and *MAD2L2* was detected using Western Blotting. **C** Plate cloning experiment showed that the number of cloned cell clusters formed by hepatocellular carcinoma cells after knockdown of *STAM, ANXA5* and *MAD2L2* was significantly reduced; **D** The results of CCK8 experiment showed that inhibiting the expression of *STAM, ANXA5* and *MAD2L2* decreased the proliferative ability of HCC cells; **E** Scratch assay showed that knockdown of *STAM, ANXA5* and *MAD2L2* inhibited the migration of hepatocellular carcinoma cells; **F** Transwell experiment showed that the migration ability of hepatocellular carcinoma cells was weakened after inhibiting the expression of *STAM, ANXA5* and *MAD2L2*.*P < 0.05, **P < 0.01, ***P < 0.001, there was no significant difference in ns

To clarify the role of exhausted CD8^+^ T cell-related genes *STAM*, *ANXA5*, and *MAD2L2* in HCC progression, we initially assessed their expression levels in normal hepatocytes and HCC cell lines using western blotting. The results revealed higher expression levels of *STAM, ANXA5*, and *MAD2L2* in liver cancer cell lines compared to normal hepatocytes (Fig. 8A). In addition, in liver cancer cell lines MHCC97H, MHCC97L, and HCCLM3, which exhibited relatively high expression levels, we knocked down *STAM, ANXA5*, and *MAD2L2* using siRNA (Fig. 8B) and observed alterations in the biological functions of HCC cells. Plate cloning and CCK8 experiments showed that the inhibition of *STAM, ANXA5*, and *M2D2L2* expression significantly reduced the clonal formation and proliferation of hepatoma cells compared to the control group (Fig. 8C, D). As shown in Fig. 8E, F, the knockdown of *STAM*, *ANXA5*, and *M2D2L2* significantly inhibited the migration ability of HCC cells.

## Discussion

This study aimed to explore the role of CD8^+^ TEX cells in hepatocellular carcinoma (HCC) and to develop a prognostic model based on their unique characteristics and molecular patterns. HCC ranks as the sixth most common malignancy and the fourth leading cause of cancer-related deaths worldwide, signifying its ongoing prominence as a major health concern [2]. Despite notable advancements in anti-HCC therapy, the long-term prognosis for patients with HCC remains unfavourable because of a limited understanding of the underlying mechanisms of tumour development and the absence of personalized treatment options for advanced HCC [23]. Existing staging systems, such as TNM staging and the Japanese comprehensive staging of liver cancer, mainly consider the influence of physical indicators, such as tumour burden, on patient prognosis. However, patients at the same stage often experience different prognoses. Risk stratification and individualized treatment for distinct patients warrant further exploration [24]. Therefore, early and precise prognosis prediction for patients with HCC has attracted considerable attention. CD8^+^ TEX cells, characterized by unique molecular patterns and transcriptional characteristics, are closely related to the occurrence and progression of HCC, making them promising diagnostic and prognostic biomarkers as well as potential therapeutic targets.

CD8^+^ T cells play a crucial role in the antitumour immune response of the body, and patients with HCC who exhibit an antigen-specific CD8^+^ T cell response tend to have better overall survival. Tumour tissue-infiltrating CD8^+^ T cells are closely associated with postoperative survival and tumour recurrence [25–27]. However, HCC has a high degree of molecular complexity and genetic heterogeneity, leading to the recognition of different tumour antigens in HCC tissues. Specific CD8^+^ T cells recognize these tumour-associated antigens in autologous tumour tissues, and this process is associated with tumour malignancy. Notably, Flecken T et al. [11] showed that specific CD8^+^ T-cell recognition of tumour-associated antigens was associated with prolonged progression-free survival in patients with HCC. Additionally, several studies have highlighted significant heterogeneity among different CD8^+^ T-cell immune responses to tumour-associated antigens within different cohorts of patients with HCC [11, 28, 29]. In the tumour microenvironment, persistent stimulation by antigens stimulation leads to the gradual loss of effector function and memory characteristics. Consequently, tumour-infiltrating CD8^+^ T cells gradually become CD8^+^ TEX cells, exhibiting reduced functionalities [6]. The proportion and surface molecular marker distribution of CD8^+^ TEX cells may vary in different tissues and diseases. However, no systematic research has explored the differences in molecular marker genes on the surface of CD8^+^ TEX cells and their correlation with the prognosis of patients with HCC.

In this study, our results revealed that the prognosis for patients with HCC in the high-risk group was notably worse compared to the low-risk group. Additionally, the risk score was identified as an independent risk factor for the prognosis of HCC patients. Differentially expressed genes in the high-risk group were mainly associated with the regulation of the immune response, the hypoxia-inducible factor-1 (HIF-1) signalling pathway, and the tumour PD-1/PD-L1 immune checkpoint pathway. This underscores the effectiveness of CD8^+^ TEX marker genes in HCC as potential targets for antitumour therapy, including immune checkpoint blockade [30]. We also identified a strong correlation between the high-risk group of HCC patients and several clinicopathological features, such as BMI, AFP level, T stage, histological grade, pathological grade, and micro-vascular invasion. This suggests that CD8^+^ TEX cells promote the malignant progression of HCC and participate in the invasive development of the HCC metastasis process. Therefore, exploring individual differences and epigenetic modifications in CD8 ^+^ TEX cells in HCC enables targeted intervention for personalized, comprehensive treatment, enhancing survival prognosis and developing more effective treatment methods.

In the present study, *MAD2L2, STAM, ANXA5, TBL1XR1, FKBP1A*, and *PPM1G* were identified as the prognostic differential genes associated with CD8^+^ TEX in HCC. Some of these genes have been studied in the context of model genes. For instance, *MAD2L2* is an important component of the mitotic checkpoint complex that interacts with various proteins and is critically involved in various cellular functions, including DNA synthesis, mitosis, and phenotypes caused by changes in cell exhaustion [31]. *STAM* participates in intracellular signal transduction, mediates DNA synthesis and growth factor signal transduction under the stimulation of IL-2 and other cytokines, and plays a role in T cell development [32]. As a member of the calcium-dependent phospholipid-binding protein family, annexin A5 plays a regulatory role in physiological and pathological processes, such as cell signal transduction, inflammation, growth, and proliferation, and is involved in tumour progression, invasion, metastasis, and drug resistance, and the treatment processes [33]. Over-expression of *ANXA5* promotes malignancy and lymph node metastasis in mouse HCC cells and is a potential marker of malignant tumours and lymph node metastasis [34]. *TBL1XR1* is a transcriptional cofactor containing F-box and WD-40 domains, involved in the regulation of various signal transduction pathways, and plays an important role in the epithelial-mesenchymal transition, drug resistance, proliferation and play an important role in metastasis [35–37]. *FKBP1A* is a member of the immunophilin protein family, which prevents TGF-*β* receptors from being activated by ligands, and mediates immune response regulation and protein folding and transport processes [38]. *PPM1G* belongs to the PP2C family of protein phosphatases, and the PP2C family is a negative regulator of cell stress response pathways. *PPM1G* also plays an important role in regulating cell cycle progression [39]. In previous studies, the biological significance of *TBL1XR1*, *FKBP1A*, and *PPM1G* in tumours has been well-documented. The present study demonstrated that the expression levels *of ANXA5, MAD2L2*, and *STAM* in liver cancer cell lines were significantly higher than those in normal hepatocytes. Moreover, the knockdown of these genes significantly inhibited the proliferation and migration of liver cancer cells, highlighting their potential influence on the malignant progression of HCC. Consequently, these CD8^+^ TEX characteristic genes emerge as promising candidates for targeted therapies in HCC.

In addition, we screened six potential small-molecule compounds: BX.795, Dasatinib, Elesclomol, BMS-754807, Doxorubicin, and Epothilone-B. BX-795, functioning as a PDK-1/TBK-1 inhibitor, blocks PDK1/Akt signalling within tumour cells, thereby inhibiting their proliferation and inducing apoptosis [40]. Dasatinib, a multi-target kinase inhibitor, inhibits the proliferation, invasion, and migration of liver cancer cells and is a potential drug for HCC targeted therapy [41]. Elesclomol, known for its safety in clinical applications, exhibits strong anti-cancer activity. Furthermore, when combined with taxane anti-cancer drugs, it improves tumour-free survival and tumour radical cure rates [42]. BMS-754807, an IGF1R/IR inhibitor, induces G2/M arrest in tumour cells and inhibits tumour cell proliferation [43]. Similarly, Doxorubicin and epothilone-B have been extensively studied as anti-tumour therapies. In the present study, patients in the low-risk group were more sensitive to BX.795, Dasatinib and Elesclomol, whereas those in the high-risk group were more sensitive to BMS.754807, doxorubicin, and epothilone. The culmination of these findings underscores the potential of these small-molecule compounds as therapeutic agents for HCC; however, further analysis is needed in the near future.

However, there are still certain limitations in this study, and there is still a lack of real-world data to validate this prediction model. In addition, there is a lack of in-depth exploration and in vivo experimental verification of the role and mechanism of CD8^+^ TEX-related gene in HCC. Therefore, in future research, we will continue to explore the mechanism of CD8^+^ TEX-related gene in HCC.

## Conclusion

This study highlights the crucial role of CD8^+^ TEX in the occurrence and progression of HCC. Leveraging the epigenetic and transcriptomic characteristics of CD8^+^ TEX, a robust prognostic model was constructed. Our findings demonstrated the model’s effectiveness in stratifying patients with HCC into high- and low-prognostic risk categories, offering a reliable tool for predicting patient outcomes. The nomogram, integrating risk scores with clinicopathological data, provides an effective and accurate means of predicting the survival of patients with HCC. These insights contribute to our understanding of HCC and provide a foundation for the development of individualized treatment plans.

### Supplementary Information

**Additional file 1.** 

**Additional file 2:** **Table 1. **Differential expression levels of CD8^+^ TEX-related genes.

**Additional file 3:** **Table 2.** Prognosis-related CD8^+^ TEX-related differentially expressed genes in HCC.

**Additional file 4:** **Table 3. **Drugs with signifcant diferences in IC50 values between high-risk and low-risk groups.

## Data Availability

The TCGA datasets presented in this study can be found in online repositories. The data used to support the findings of this study are available from the corresponding author upon request.

## Abbreviations

HCC: Hepatocellular Carcinoma
CD8^+^ TEX: CD8^+^ T Cell Exhaustion
COX: Cox Regression
LASSO: Least Absolute Shrinkage and Selection Operator
AFP: Alpha-Fetoprotein
TNM: Tumor-Node-Metastasis
GDSC: Cancer Drug Sensitivity Genomics
PDK-1: Phosphoinositide-Dependent Kinase-1
IGF1R/IR: Insulin-Like Growth Factor 1 Receptor/Insulin Receptor
siRNA: Small Interfering RNA
