# Supplementary material for: Identification of a Novel CD8+ T cell exhaustion-related gene signature for predicting survival in hepatocellular carcinoma
Source: BMC Cancer. 2023 Dec 4;23:1185. doi: 10.1186/s12885-023-11648-x (PMC10694949; doi:10.1186/s12885-023-11648-x)
Supplement: Supplementary file 1 — Additional file 1. [file 12885_2023_11648_MOESM1_ESM.pdf]

Figure 8. Expression of *STAM, ANXA5* and *MAD2L2* in HCC cell lines.(A) The expressions of *STAM, ANXA5* and *MAD2L2* in normal hepatocytes and hepatoma cell lines were detected using Western blotting. Knocking down *STAM, ANXA5* and *MAD2L2* inhibited the proliferation and migration of HCC cells.

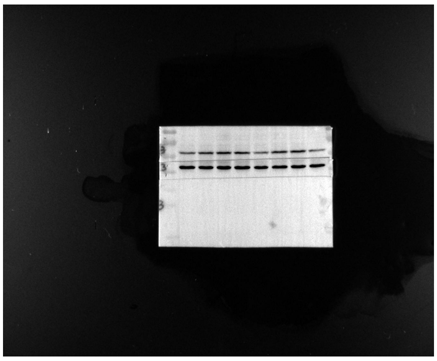
(*STAM*)

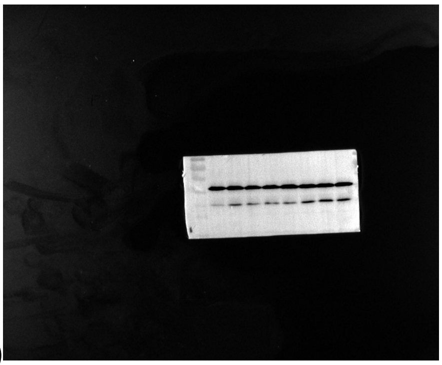
(*ANXA5*)

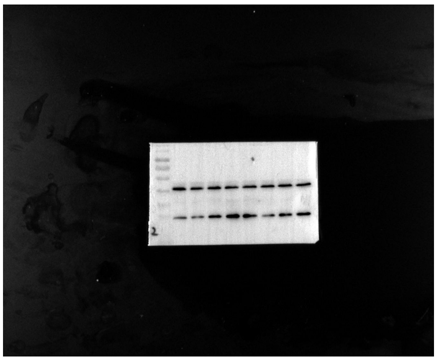
(*MAD2L2*)

(B) The knockdown efficiency of *STAM, ANXA5* and *MAD2L2* was detected using Western Blotting.

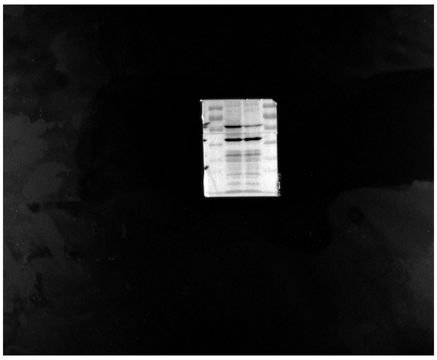
(*STAM*)

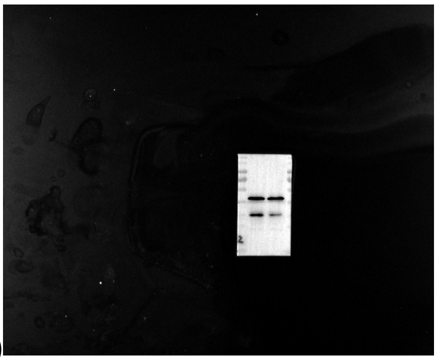
(*ANXA5*)

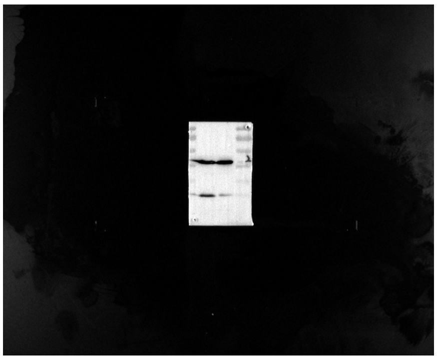
(*MAD2L2*)
